# Past and present experience shifts audiovisual temporal perception in rats

**DOI:** 10.3389/fnbeh.2023.1287587

**Published:** 2023-10-16

**Authors:** Mohammed U. Al-youzbaki, Ashley L. Schormans, Brian L. Allman

**Affiliations:** Department of Anatomy and Cell Biology, Schulich School of Medicine and Dentistry, Western University, London, ON, Canada

**Keywords:** rapid recalibration, rat, audiovisual temporal perception, temporal order judgment, synchrony judgment

## Abstract

Our brains have a propensity to integrate closely-timed auditory and visual stimuli into a unified percept; a phenomenon that is highly malleable based on prior sensory experiences, and is known to be altered in clinical populations. While the neural correlates of audiovisual temporal perception have been investigated using neuroimaging and electroencephalography techniques in humans, animal research will be required to uncover the underlying cellular and molecular mechanisms. Prior to conducting such mechanistic studies, it is important to first confirm the translational potential of any prospective animal model. Thus, in the present study, we conducted a series of experiments to determine if rats show the hallmarks of audiovisual temporal perception observed in neurotypical humans, and whether the rat behavioral paradigms could reveal when they experienced perceptual disruptions akin to those observed in neurodevelopmental disorders. After training rats to perform a temporal order judgment (TOJ) or synchrony judgment (SJ) task, we found that the rats’ perception was malleable based on their past and present sensory experiences. More specifically, passive exposure to asynchronous audiovisual stimulation in the minutes prior to behavioral testing caused the rats’ perception to predictably shift in the direction of the leading stimulus; findings which represent the first time that this form of audiovisual perceptual malleability has been reported in non-human subjects. Furthermore, rats performing the TOJ task also showed evidence of rapid recalibration, in which their audiovisual temporal perception on the current trial was predictably influenced by the timing lag between the auditory and visual stimuli in the preceding trial. Finally, by manipulating either experimental testing parameters or altering the rats’ neurochemistry with a systemic injection of MK-801, we showed that the TOJ and SJ tasks could identify when the rats had difficulty judging the timing of audiovisual stimuli. These findings confirm that the behavioral paradigms are indeed suitable for future testing of rats with perceptual disruptions in audiovisual processing. Overall, our collective results highlight that rats represent an excellent animal model to study the cellular and molecular mechanisms underlying the acuity and malleability of audiovisual temporal perception, as they showcase the perceptual hallmarks commonly observed in humans.

## 1. Introduction

How we perceive the world around us is greatly influenced by our brain’s ability to process and integrate information from our different senses. The relative timing of auditory and visual events within the environment not only influences the degree of multisensory integration that occurs, but ultimately impacts how these stimuli are perceived by the observer. In this regard, it is well established that the brain has a propensity to merge closely-timed auditory and visual stimuli into a unified percept, even when the two stimuli occur at separate moments in time. To investigate the brain regions and neural activity associated with this audiovisual temporal perception, past studies have used functional neuroimaging and electroencephalography (EEG) techniques while participants were asked to make perceptual judgments regarding the timing of auditory and visual stimuli (Adhikari et al., 2013; Binder, 2015; Basharat et al., 2018; Love et al., 2018; Simon et al., 2018; Zhou et al., 2020; Johnston et al., 2022; Zhou et al., 2023). While much has been learned from these non-invasive studies, the cellular and molecular mechanisms that support the neural correlates of audiovisual temporal perception remain elusive, in large part because studying these underlying mechanisms requires more manipulative and invasive approaches like those available in animal research. Ultimately, there are clinical implications for uncovering the cellular and molecular mechanisms of audiovisual temporal perception as a variety of clinical populations can experience difficulty detecting subtle timing lags between auditory and visual stimulation (for review, see Zhou et al., 2018; Meilleur et al., 2020).

Looking forward, if animals are to be used to eventually determine the mechanisms underlying the neural correlates of audiovisual temporal perception, we suggest that there are important initial experiments that should be conducted to first verify the suitability of the animal model. For example, researchers should confirm that the chosen animal species (e.g., rats) can be tested using behavioral tasks of audiovisual temporal perception that are consistent with those performed by humans, and that both species display similar performance metrics. Moreover, like human participants, the rats should show evidence that their audiovisual temporal perception can be predictably altered by their prior sensory experience, as well as by changes made to the stimulation parameters in their testing paradigms. Finally, the behavioral tasks should be capable of revealing when the rats have perceptual difficulty judging the timing of the auditory and visual stimuli, as this will be important in future studies that seek to use rats to study clinically-relevant disruptions in audiovisual temporal perception. In the next sections, we summarize the human literature that provides the rationale for the four experimental series in the present study; all of which were conducted with the goal of determining whether rats represent a suitable animal model to study the mechanistic basis of audiovisual temporal perception and its disruption in clinical conditions.

In humans, audiovisual temporal perception has been investigated with behavioral tasks that ask participants to judge either the temporal order or the synchrony of closely-timed auditory and visual stimuli. When performing a temporal order judgment (TOJ) task participants are presented with auditory and visual stimuli with various timing lags, and are asked to report which stimulus they perceived came first or which came second (Spence et al., 2001; Stone et al., 2001; Zampini et al., 2005a; Vatakis et al., 2008b; Keetels and Vroomen, 2012; Binder, 2015; Love et al., 2018; Takeshima, 2021). During a synchrony judgment (SJ) task, participants are presented with auditory and visual stimuli with various timing lags between the stimuli, and asked to report whether they perceived the stimuli to have occurred at the same moment in time or not (Zampini et al., 2005a; van Eijk et al., 2008; Vatakis et al., 2008b; Keetels and Vroomen, 2012; O’Donohue et al., 2022; Ainsworth and Bertone, 2023). Although animal researchers have developed TOJ tasks and a modified version of a SJ task for rats (Schormans et al., 2017; Mafi et al., 2023; Paulcan et al., 2023), no prior studies have confirmed that rats can be trained to perform a full version of the SJ task, with performance metrics akin to humans. In the present study, we first designed such an SJ task for rats, and then investigated if they show experience-dependent shifts in their audiovisual temporal perception during performance of both the SJ and TOJ tasks.

A hallmark of audiovisual temporal perception in humans is its malleability based on the participant’s past and present sensory experiences. For example, when participants are repeatedly exposed to asynchronous audiovisual stimuli (e.g., the visual stimulus precedes the auditory stimulus by ~200 ms) for a few minutes prior to the testing session, their audiovisual temporal perception shifts, as they are now more likely to judge such stimuli pairings as being synchronous (Fujisaki et al., 2004; Vroomen et al., 2004; Navarra et al., 2005; Vatakis et al., 2008b; Roseboom and Arnold, 2011; Heron et al., 2012; Roseboom et al., 2013; O’Donohue et al., 2022). On a more rapid time scale, perceptual malleability can also be experienced on a trial by trial basis during a given testing session, such that the participants’ perception of whether an auditory and visual stimulus pair was synchronous/asynchronous is recalibrated according to the temporal offset of the stimuli in the preceding trial (Van der Burg et al., 2013; Van der Burg and Goodbourn, 2015; Van der Burg et al., 2015b; Simon et al., 2017, 2018; Takeshima, 2021). Furthermore, previous studies have confirmed that performance of the TOJ and SJ tasks can be significantly influenced by experimental parameters, such as the intensity and duration of the stimuli (Boenke et al., 2009; Krueger Fister et al., 2016) as well as the overall task conditions (Zampini et al., 2005a,b; Stevenson and Wallace, 2013). Importantly, we used the experimental approaches outlined in the aforementioned human studies as the basis to design experiments for rats that would allow us to assess whether they, too, experience malleability of their audiovisual temporal perception.

In the present study, we trained rats to perform the newly-designed SJ task or a previously established TOJ task (Schormans et al., 2017) to investigate if their audiovisual temporal perception would shift following repeated exposure to asynchronous audiovisual stimuli in the minutes prior to testing (Experimental Series 1). Next, we determined whether rats, like humans, experience rapid recalibration of their audiovisual temporal perception as a consequence of the timing lag between the auditory and visual stimuli in the preceding trial (Experimental Series 2). In Experimental Series 3, we assessed the consequence of having the rats perform the TOJ and SJ tasks in the presence of a competing background noise, as this would allow us to determine the extent that their audiovisual temporal perception was sensitive to manipulation of the task parameters. Finally, we were motivated to test whether the rat versions of the TOJ and SJ tasks were robust to perceptual disruptions akin to those observed in various clinical populations. Compared to neurotypical subjects, individuals with neurodevelopmental disorders such as schizophrenia and ASD can experience challenges detecting slight timing differences in auditory and visual stimuli, resulting in a decreased acuity of their audiovisual temporal perception (for review, see Zhou et al., 2018; Meilleur et al., 2020). While the mechanisms underlying the sensory processing and perceptual alterations in schizophrenia and ASD are not fully resolved, it has been suggested that disruption in glutamatergic neurotransmission likely plays a significant role (Gonzalez-Burgos and Lewis, 2012; Canitano and Pallagrosi, 2017). Thus, in the present study, we reasoned that if the rat versions of the TOJ and SJ tasks are indeed suitable for future mechanistic studies, it would be important to first confirm that it was possible to identify when the rats have perceptual difficulty judging the timing of the auditory and visual stimuli. To that end, in Experimental Series 4, rats were tested on the TOJ and SJ tasks to assess their audiovisual temporal perception after their neurochemistry was altered by a systemic injection of MK-801; a drug that has been shown to impair other forms of sensory processing and perception by disrupting glutamatergic neurotransmission through the antagonism of the N-Methyl-D-aspartate (NMDA) receptor (Paine and Carlezon, 2009; Sivarao et al., 2013; Parras et al., 2020; Onofrychuk et al., 2021; Janz et al., 2022; Patrono et al., 2023).

Overall, we hypothesized that the rats would show evidence of malleability of their audiovisual temporal perception based on their past and present experience (Experimental Series 1 & 2, respectively). Furthermore, we predicted that the rats’ ability to detect subtle timing lags between the auditory and visual stimulation would be negatively affected by manipulating the auditory stimulation parameters during testing (Experimental Series 3) or by pharmacologically disrupting their neurochemistry (Experimental Series 4). Taken together, our collective results confirm that rats demonstrate the hallmarks of audiovisual temporal perception observed in humans; findings which support the future use of rats to investigate the cellular and molecular mechanisms that underlie the acuity and malleability of audiovisual temporal perception.

## 2. Methods

### 2.1. Animals

Overall, the present study consisted of four experimental series conducted in animals trained to perform a previously-established TOJ task (Schormans et al., 2017) or a newly-developed SJ task. In total, 23 adult male Sprague–Dawley rats (Charles River Laboratories, Inc., Wilmington, MA, United States) were used across all four experimental series (described in detail below), with the majority of rats being tested in each experimental series. Rats were housed on a 12-h light–dark cycle with food and water *ad libitum* unless otherwise stated. All experimental procedures were approved by the University of Western Ontario Animal Care and Use Committee and were in accordance with the guidelines established by the Canadian Council of Animal Care.

### 2.2. Behavioral apparatus and audiovisual stimuli

**Figure 1:**
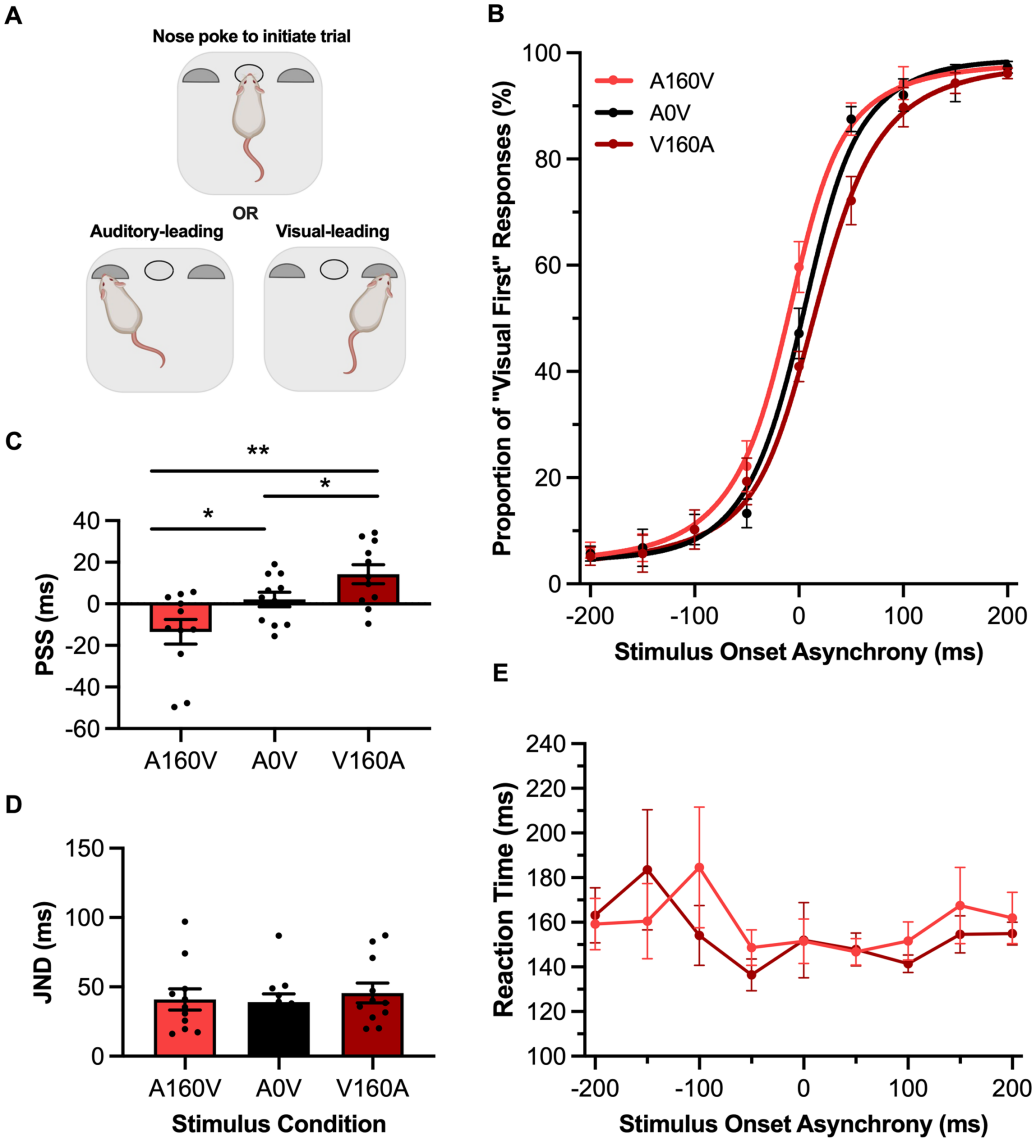
Passive sensory exposure shifted perception while sparing audiovisual temporal acuity in rats performing the TOJ task. **(A)** Schematic overview of the rat temporal order judgment (TOJ) task within the operant chamber (created with BioRender.com). **(B)** Behavioral performance of Sprague Dawley rats (*n* = 10) on a TOJ task plotted as proportion of “visual first” trials. Rats were exposed to either synchronous (A0V, black), 160 ms auditory-leading (A160V, red), or 160 ms visual-leading (V160A, dark red) stimuli prior to behavioral testing. **(C)** The point of subjective simultaneity (PSS) was extracted from the three fitted curves A160V, A0V, V160A. Consistent with humans, the PSS was shifted in the direction of the prior sensory experience. **(D)** The just noticeable difference (JND) of the three fitted curves did not change in response to the three passive exposure conditions. **(E)** Reaction time of rats for each SOA for the auditory-leading (A160V; red) and visual-leading (V160A; dark red) prior sensory experiences. Values are presented as mean ± SEM for PSS and JND, *p < 0.05, **p < 0.017.

**Figure 2:**
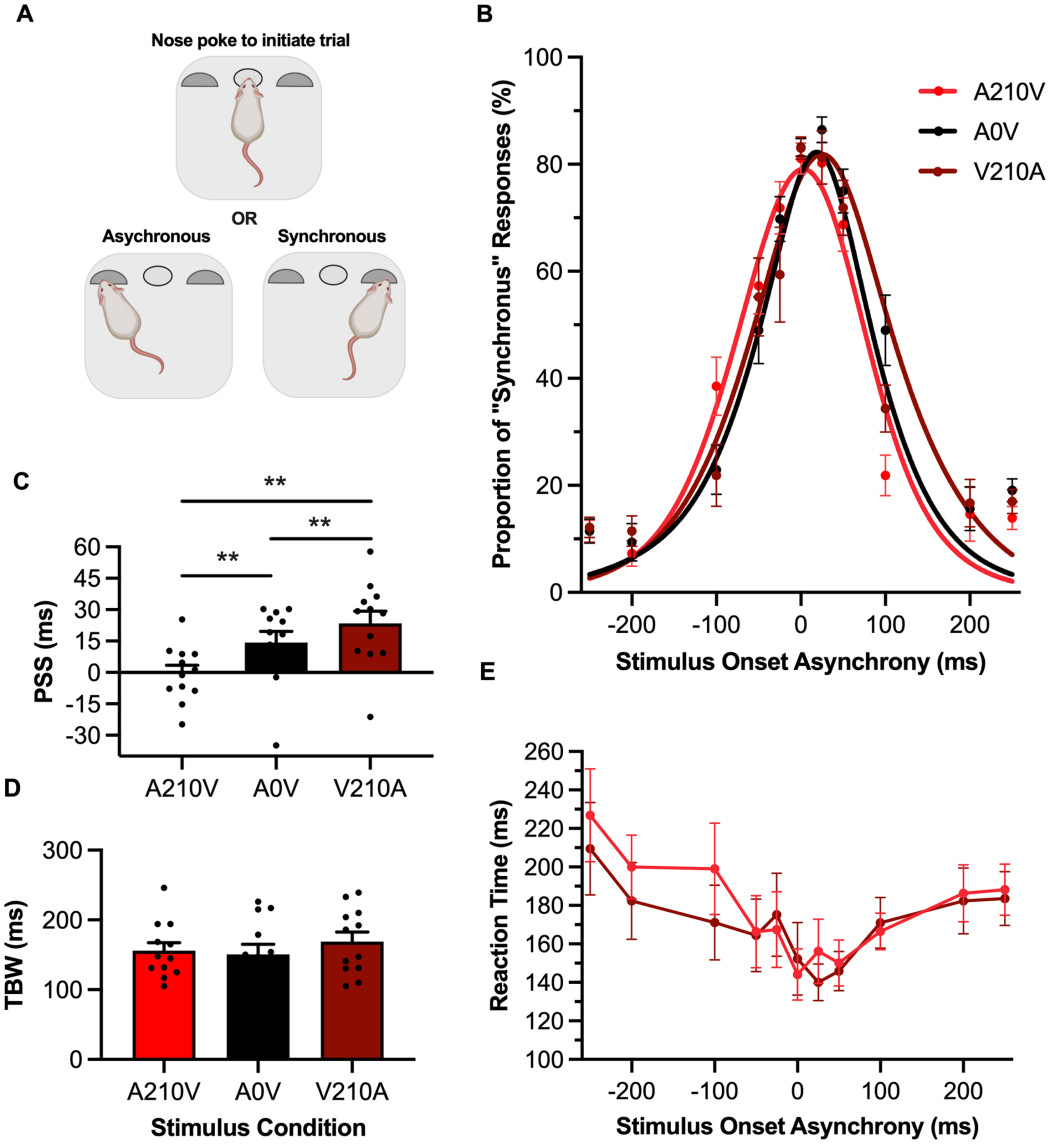
Passive sensory exposure shifted perception while sparing audiovisual temporal acuity in rats performing the SJ task. **(A)** Schematic overview of the rat synchrony judgment (SJ) task within the operant chamber (created with BioRender.com). **(B)** Behavioral performance of Sprague Dawley rats (*n* = 12) on an SJ task plotted as proportion of “synchronous” trials. Rats were exposed to either synchronous (A0V, black), 210 ms auditory-leading (A210V, red), or 210 ms visual-leading (V210A, dark red) stimuli prior to behavioral testing. **(C)** The point of subjective simultaneity (PSS) was extracted from the three fitted curves A210V, A0V, V210A. Consistent with humans, the PSS was significantly shifted in the direction of the prior sensory experience. **(D)** The temporal binding window (TBW) of the three fitted curves did not change in response to the three passive exposure conditions. **(E)** Reaction time of rats for each SOA for the auditory-leading (A210V; red) and visual-leading (V210A; dark red) prior sensory experiences. Values are presented as mean ± SEM for PSS and TBW, *p < 0.05, **p < 0.017.

Using appetitive operant conditioning, rats were trained on a two-alternative forced-choice paradigm to perform either an audiovisual TOJ task or SJ task. For the TOJ task, rats were appetitively conditioned to differentiate between auditory-first or visual-first audiovisual stimuli and report which modality was presented first in each trial (Figure 1A), whereas rats trained on the SJ task were appetitively conditioned to report if they perceived the auditory and visual stimuli to have been presented at the same moment in time (i.e., synchronous) or at different times (i.e., asynchronous) (Figure 2A). For both tasks, behavioral training began at 70 days old (body mass: 265 ± 13 g), and the rats were trained 6–7 days a week.

Behavioral training and testing were carried out in a standard modular test chamber (ENV-008CT; Med Associates Inc., St. Albans, VT) which was located within a sound-attenuating enclosure (Med Associates Inc.). The standard modular test chamber was illuminated by a house light located on the back wall, whereas the front wall contained a center nose-poke port, a left feeder trough and a right feeder trough; all three of which are equipped with an infrared (IR) detector to monitor the rat’s performance. The behavioral chamber was interfaced with real-time processing hardware (RZ6 and BH-32; Tucker Davis Technologies, Alachua, FL). Custom behavioral protocols were run in MATLAB (EPsych Toolbox, dstolz.github.io/epsych/) to monitor nose pokes, control the presentation of auditory and visual stimuli, and deliver positive reinforcement (i.e., a food pellet) or punishment (i.e., turning off the house light and an inability to initiate the next trial for 15 s). Stimulus information (i.e., intensity, duration, timing, etc.), feeder choice, and response duration were saved for each trial using custom MATLAB scripts (EPsych Toolbox, dstolz.github.io/epsych/).

The auditory stimulus was a 75 dB SPL noise burst (50 ms; 1–32 kHz) presented from a speaker (FT28D, Fostex, Tokyo) mounted on the ceiling of the behavioral chamber near the front wall. The auditory stimulus intensity was calibrated using a custom MATLAB software with a 1/4-inch microphone (2,530, Larson Davis, Depew, NY) and preamplifier (2,221; Larson Davis). The visual stimulus was a 27-lux light flash (50 ms) from an LED (ENV-229 M; Med Associates Inc.) located above the center nose poke. An LED light meter (model LT45, Extech Instruments, Nashua, NH) was used to determine the intensity of the visual stimulus.

### 2.3. Behavioral training for the TOJ and SJ tasks

Prior to beginning training, rats were weighed daily and maintained on a food restricted diet until reaching approximately 90% of their free-feeding body mass. Over several training phases, the rats learned to associate a given auditory–visual stimulus pairing with a specific feeder trough (i.e., TOJ task: visual-first = right trough and auditory-first = left trough; SJ task: synchronous = right trough and asynchronous = left trough). The first training phase consisted of the rats habituating to the behavioral chamber for 30 min/day for 3–4 days. During this phase, spontaneous nose pokes into the center port resulted in the immediate presentation of an audiovisual stimulus (TOJ: ±400 ms, SJ: 0 ms or ± 400 ms) and the delivery of a positive reinforcement (45 mg food pellet, Bio-Serv, Frenchtown, NJ, United States) to the feeder trough associated with the presented stimulus. It is important to note that positive value stimulus onset asynchronies (SOAs) (e.g., +400 ms) refer to the visual-leading stimulus, whereas negative value SOAs (e.g., −400 ms) correspond to trials with an auditory-leading stimulus. In this habituation phase, a second pellet was also delivered if the animal went to the correct feeder trough, as this would provide an associative cue between the given audiovisual stimulus and the correct feeder trough.

Following the habituation phase, the first pellet was removed so the rats would only be positively reinforced if they chose the correct feeder trough following the presentation of an audiovisual stimulus. The stimulus onset asynchrony (SOA) remained at 400 ms for both the TOJ and SJ tasks. During each daily training session, rats initiated a trial by holding their nose in the center port, which led to the presentation of an audiovisual stimulus after a variable amount of time (range: 1.5 to 4 s). Correct trials were positively reinforced with the delivery of a food pellet, and incorrect responses resulted in the house light turning off for 15 s and the inability to initiate the next trial. As training progressed and performance criterions were reached (i.e., 5 consecutive days at >80% correct), the SOA was eventually decreased to ±200 ms for the TOJ task and ± 250 ms for the SJ task.

### 2.4. Behavioral testing and analysis

Once the rats reached the final training criterion for the TOJ task (±200 ms SOA) and SJ task (±250 ms SOA), experimental test sessions were introduced to assess their audiovisual temporal perception across a range of SOAs. Experimenters were not blinded to the different testing conditions or the data analysis. To reduce the potential of the rats developing a side bias during the experimental test sessions, 70% of the trials were the same as the training stimuli (i.e., TOJ: ± 200 ms; SJ: 0, ± 250 ms) and the remaining trials consisted of the random presentation of novel SOAs (i.e., TOJ: 0, ±50, ±100, ±150 ms; SJ: ±25, ±50, ±100, ±200 ms). Unique to the experimental test sessions, the novel SOAs were reinforced with food pellets, regardless of the outcome of the trial. In contrast, the trained stimulus conditions were positively reinforced for correct responses and punished for incorrect responses with a 15-s timeout.

During test sessions of the TOJ task, the rats’ performance across the range of SOAs was quantified as the proportion of trials perceived as visual-first (i.e., right feeder trough response). The data collected during the TOJ task were individually fit for each rat’s test session using the maximum-likelihood procedure of the open-source package psignifit 4 for MATLAB (Schütt et al., 2016). Each distribution was fit using a cumulative gaussian function with free parameters of threshold (i.e., the level at which the unscaled sigmoid function has a value of 0.5) and width (i.e., the difference between the levels at which the function reaches 0.05 and 0.95) (Schütt et al., 2016). In the present study, it was important that we assessed the rats’ performance metrics during the TOJ task that are consistent with human studies. For example, based on the psychometric curve derived from a participant’s TOJ task performance, it is possible to calculate their point of subjective simultaneity (PSS), which describes the actual timing of the auditory and visual stimuli when the participant was most unsure of the temporal order. Also calculated from the TOJ task is the participant’s just noticeable difference (JND), which represents the smallest interval of time between the separately presented auditory and visual stimuli that they could reliably detect (Vatakis et al., 2008a; Vroomen and Stekelenburg, 2011; Keetels and Vroomen, 2012). Thus, in the present study, the point of subjective simultaneity (PSS) in the rat TOJ task was determined through the SOA at which 50% of the responses were judged to be ‘visual-first’, and the just noticeable difference (JND) was determined by the boundaries of 25 to 75% visual-first responses and then divided by two.

During test session of the SJ task, the rats’ performance across the range of SOAs was quantified as the proportion of trials perceived as synchronous (i.e., right feeder trough response). Consistent with previous analyses of data from an SJ task performed by humans (Simon et al., 2018), a simple-term gaussian psychometric fitting function was used with free parameters for mean, standard deviation, and amplitude (MATLAB fit.m). In parity with past human studies, we analyzed each rat’s psychometric curve to determine their PSS, which in the case of the SJ task represents the audiovisual SOA with the highest chance of being perceived as synchronous. Moreover, consistent with human studies, we also calculated the rats’ temporal binding window (TBW); i.e., the epoch of time over which they had a high likelihood of perceiving the auditory and visual stimuli as being synchronous, even when it was not (Wallace and Stevenson, 2014). Thus, the PSS of the rat SJ task was determined by the peak of the fitted curve, and the temporal binding window (TBW) was determined by the two boundaries at the 50% perceived synchronous trials. It is important to note that the TBW calculated from the SJ task, as well as the JND metric derived from the TOJ task, can be used to characterize the temporal acuity of a participant’s audiovisual perception (i.e., their sensitivity to detect subtle timing lags between the auditory and visual stimulation).

In addition to examining the rats’ performance during each experimental series, their reaction time was also measured. Using an IR detector in the center nose-poke port, reaction time was measured as the epoch of time between the onset of the stimulus presentation and when the rat removed its nose from the center port. Mean reaction time across each SOA in the TOJ and SJ tasks were calculated using custom MATLAB scripts.

### 2.5. Experimental series 1: investigating the malleability of audiovisual temporal perception following passive exposure to asynchronous stimuli

A protocol consistent with past human studies (Fujisaki et al., 2004; Vroomen et al., 2004; Harrar and Harris, 2008) was used to investigate whether the rats’ audiovisual temporal perception would predictably shift following repeated exposure to asynchronous audiovisual stimuli in the minutes prior to testing. Rats were repeatedly presented with an audiovisual stimulus for 6 min prior to experimental testing in the TOJ task (*n* = 10 rats; 8–9 months old) and SJ task (*n* = 12 rats; 9–10 months old). The passive sensory exposure was carried out in a different chamber and sound-attenuating enclosure (Med Associates Inc.) than those used for behavioral testing. Using custom MATLAB protocols and real-time processing hardware (RZ6 and BH-32; Tucker Davis Technologies, Alachua, FL), the auditory stimulus (75 dB SPL noise burst; 50 ms; 1–32 kHz) was presented from a speaker (FT28D, Fostex, Tokyo) mounted on the ceiling of the chamber near the front wall, whereas the visual stimulus (27-lux, 50 ms light flash) was presented from an LED (ENV-229 M; Med Associates Inc.) located at the front of the chamber.

For the passive sensory exposure, the auditory and visual stimulus pairing was presented as either synchronous (0 ms offset), auditory-leading (TOJ: −160 ms; SJ: −210 ms), or visual-leading (TOJ: +160 ms; SJ: +210 ms). Thus, the SOAs of the asynchronous stimuli conditions used in the passive exposure were 40 ms shorter than the TOJ and SJ testing extremes (i.e., TOJ: ±200 ms; SJ: ±250 ms) and were selected based on pilot testing. Each audiovisual stimulus was presented 360 times with an inter-trial interval of 800 to 1,200 ms and the protocol lasted for a total of 6 min. Immediately following the passive sensory exposure, rats underwent a TOJ or SJ test session, as described above. For each rat, the order of the passive exposure conditions (i.e., synchronous, auditory-leading or visual-leading) was randomized and counter-balanced across all rats.

To assess the behavioral results, a custom MATLAB script was used to analyze the first 160 trials for each individual test session, as pilot testing revealed no effect of passive exposure in the last 10 min of the experimental test sessions. For both the TOJ and SJ tasks, the behavioral testing protocol and function fitting of the psychometric curves were carried out in the same manner as described above. To assess the possibility of perceptual malleability following the passive exposure protocols, we determined whether there was a shift in the rats’ PSS between the synchronous exposure condition (A0V) versus the two asynchronous exposure conditions when tested with the TOJ task (auditory-leading: A160V; visual-leading: V160A) or the SJ task (auditory-leading: A210V; visual-leading: V210A). Furthermore, to determine if the passive exposure affected the rats’ temporal acuity, we compared their JND (TOJ task) or TBW (SJ task) derived from the test sessions that followed the three passive exposure conditions. The rats’ reaction times during the various test sessions were also tabulated.

### 2.6. Experimental series 2: assessment of single-trial recalibration as evidence of rapid adaptation of the rats’ audiovisual temporal perception

To assess whether rats experience rapid recalibration of their audiovisual temporal perception as a consequence of the timing lag between the auditory and visual stimuli in the preceding trial, they underwent three experimental test sessions to ensure enough trials were performed at each SOA. For the TOJ task (*n* = 11 rats; 9–10 months old) or SJ task (*n* = 12 rats; 6–8 months old), each rat performed a minimum of 60 or 30 trials of each novel SOA, respectively. Using custom MATLAB scripts, trials for each test session were split into two categories: (1) trials that followed a visual-leading stimulus, and (2) trials that followed an auditory-leading stimulus. The trials that followed a synchronous audiovisual stimulus (i.e., 0 ms SOA) were not included in the analysis. Data associated with the preceding auditory-leading and visual-leading trials were individually fit, and using the aforementioned calculations, the rats’ temporal malleability (shift in PSS) and acuity (JND or TBW) were determined from the psychometric curves corresponding to two trial types. Furthermore, the reaction times associated with the various SOAs were compared between those trials that followed auditory-leading vs. visual-leading trials. Finally, guided by a previous human study (Van der Burg et al., 2013), a linear regression (Pearson’s correlation) was conducted to assess the potential relationship between the rapid shift in the rats’ perception (i.e., visual-leading PSS minus auditory-leading PSS) and their temporal acuity (i.e., JND in TOJ task; TBW in SJ task).

### 2.7. Experimental series 3: investigating the effect of a decrease in the signal-to-noise ratio of auditory stimulation on the rats’ audiovisual temporal perception

Rats performed the TOJ task (*n* = 10 rats; 11–12 months old) or SJ task (*n* = 8 rats; 15–16 months old) in the presence of a background noise to determine whether decreasing the signal-to-noise ratio of the auditory stimulation would ultimately affect the temporal acuity of their audiovisual perception. These experiments were conducted in the same test chamber used for the aforementioned audiovisual temporal perceptual testing, with the addition of a second speaker (FT28D, Fostex, Tokyo) mounted on the back wall. Throughout separate test sessions, the rats performed the TOJ and SJ tasks in the presence of a continuous background noise (60 dB SPL; 1–32 kHz) or quiet (i.e., no background noise presented; which served as our control). Test sessions were randomized across rats to ensure that the observed changes were not due to the testing order. Overall, the behavioral testing protocol and function fitting of the psychometric curves were carried out in the same manner as described above, thereby providing a comparison of the performance measurements (i.e., PSS; JND or TBW; reaction times) calculated from the two testing conditions (i.e., the presence vs. absence of background noise). Lastly, consistent with a previous study on humans (Stevenson and Wallace, 2013), the bounds of the JND (TOJ task) as well as the left and right side of the TBW (SJ task) were plotted to test whether decreasing the signal-to-noise ratio of the auditory stimulation would cause an asymmetry in the rats’ psychometric curve indicative of an auditory-specific effect on their temporal acuity.

### 2.8. Experimental series 4: investigating the consequence of a disruption in glutamatergic neurotransmission on the temporal acuity of audiovisual perception

We assessed the effect of disrupting glutamatergic neurotransmission on the rats’ performance of the TOJ task (*n* = 10 rats; 10–11 months old) or SJ task (*n* = 10 rats; 11–12 months old) by administering a subcutaneous injection of the NMDA receptor antagonist, dizocilpine (MK-801; 0.1 mg/kg) or saline (1 mL/kg; to serve as a control) 25 min prior to task performance. The injection order was randomized and counter-balanced across all rats. Using the experimental approaches described above, each rat’s temporal acuity was gleaned from its fitted psychometric curve, and the bounds of the JND (TOJ task) as well as the left and right side of the TBW (SJ task) were compared between the treatment conditions to ultimately examine whether the disruption to glutamatergic neurotransmission caused any asymmetries in the rats’ psychometric curves.

### 2.9. Statistics

Depending on the comparisons of interest, a variety of statistical analyses were performed, including one-way and a two-way repeated-measures analysis of variance (ANOVA), as well as paired samples *t*-tests. A repeated-measures ANOVA was selected because the measurements were performed in the same rats across each testing condition. The repeated-measures ANOVA assumes that the data are normally distrubuted and that the variances of the difference are equal between all the related groups (i.e., sphericity). As such, tests of normality and sphericity were performed prior to conducting the ANOVAs. If Mauchly’s test of sphericity was violated, the Greenhouse–Geisser correction was used. The level of statistical significance was set to *α* = 0.05 and Bonferroni post-hoc corrections were performed for multiple comparisons. Details for each statistical test are included in the Results section. GraphPad Prism (Version 9, GraphPad software Inc.) generated the graphical displays, and SPSS (Version 26, IBM Corporation) software was used for all statistical analyses. Data within the text and figures are presented as mean values ± standard error of the mean (SEM).

## 3. Results

### 3.1. Passive exposure to asynchronous stimulation predictably shifted the rats’ audiovisual temporal perception

To investigate whether the rats’ temporal perception of audiovisual stimuli would shift in response to their past sensory experience, we passively exposed them to three different stimulus conditions for 6 min prior to performing the TOJ or SJ task. More specifically, rats trained on the TOJ task were exposed to either a synchronous audiovisual stimulus (A0V), an asynchronous stimulus pair where the auditory led the visual by 160 ms (A160V) or an asynchronous stimulus pair where the visual led the auditory by 160 ms (V160A). Following this passive exposure, TOJ rats underwent an experimental test session, and their data were fit with a sigmoidal function and plotted (Figure 1B). As predicted, a one-way repeated-measures ANOVA found a significant main effect of PSS [*F* (2,20) = 13.26, *p* < 0.001, *ηp*^2^ = 0.57] and Bonferroni-adjusted paired samples *t*-tests revealed that when rats were passively-exposed to an auditory-leading stimulus their PSS was significantly shifted towards the auditory-leading side (A160V: −13.5 ± 5.9 ms vs. A0V: 2.1 ± 3.5 ms, *p* = 0.01), whereas when they were passively exposed to a visual-leading stimulus, their PSS shifted towards the visual-leading side (V160A: 14.3 ± 5.4 ms vs. A0V: 2.09 ± 3.5 ms, *p* = 0.04) (Figure 1C). Thus, the rats’ perception predictably shifted in the direction of the leading stimulus in the passive exposure; evidence that their audiovisual temporal perception was indeed malleable to their prior sensory experience. Not surprisingly based on a previous study on humans (Harrar and Harris, 2008), the aforementioned shifts in PSS were not accompanied by a change in the rats’ temporal acuity, as their JND values were unchanged between the passive exposure conditions [F (2,20) = 0.34, *p* = 0.71, *ηp*^2^ = 0.03, one-way repeated-measures ANOVA] (Figure 1D). Furthermore, there was no difference between the rats’ reaction times when they were passively exposed to the asynchronous audiovisual stimuli (i.e., two-way repeated-measures ANOVA: no main effect of exposure, [*F* (1, 10) = 0.02, *p* = 0.90, *ηp*^2^ = 0.002]; no interaction between exposure & SOA, [*F* (3.3, 33.0) = 0.72, *p* = 0.72, *ηp*^2^ = 0.07]) (Figure 1E).

Prior to the SJ task, rats were passively exposed for 6 min to either a synchronous audiovisual stimulus (A0V), an asynchronous stimulus pair where the auditory led the visual by 210 ms (A210V) or an asynchronous stimulus pair where the visual led the auditory by 210 ms (V210A). Following this passive exposure, the rats performed the SJ task, and their data were fit with a gaussian function and plotted (Figure 2B). Similar to the TOJ task results, passive exposure prior to the SJ task caused a shift in the rats’ PSS in the direction of the asynchronous stimulus pairing [*F* (2,22) = 17.35, *p* < 0.001, *ηp*^2^ = 0.61, one-way repeated-measures ANOVA] (Figure 2C). More specifically, when the rats were passively exposed to the auditory-leading stimulus, their PSS significantly shifted towards the auditory-leading (i.e., left) side of the curve in comparison to the synchronous exposure (Bonferroni-adjusted paired samples *t*-test: A210V: −0.5 ± 3.8 ms vs. A0V: 14.2 ± 5.3 ms, *p* = 0.01). Similarly, the visual-leading exposure resulted in a significant shift of the PSS towards the visual-leading (i.e., right) side when compared to the synchronous exposure (Bonferroni-adjusted paired samples *t*-test: V210A: 23.4 ± 5.8 ms vs. A0V: 14.2 ± 5.3 ms, *p* = 0.01). Ultimately, the passive exposure predictably shifted the rats’ psychometric curves during the SJ task, and this prior sensory experience did not affect the rats’ temporal acuity, as their TBW values did not differ between the passive exposure conditions [*F* (2,22) = 0.69, *p* = 0.51, ηp^2^ = 0.06, one-way repeated-measures ANOVA] (Figure 2D). Furthermore, the rats’ reaction times did not differ following the passive exposure to the asynchronous audiovisual stimuli (i.e., two-way repeated-measures ANOVA: no main effect of exposure, [*F* (1,11) = 1.17, *p* = 0.30, *ηp*^2^ = 0.10]; no interaction between exposure & SOA, [*F* (3.9, 43.2) = 1.43, *p* = 0.24, *ηp*^2^ = 0.12]) (Figure 2E).

### 3.2. Rapid recalibration of the rats’ audiovisual temporal perception was evident in the TOJ task but not SJ task

**Figure 3:**
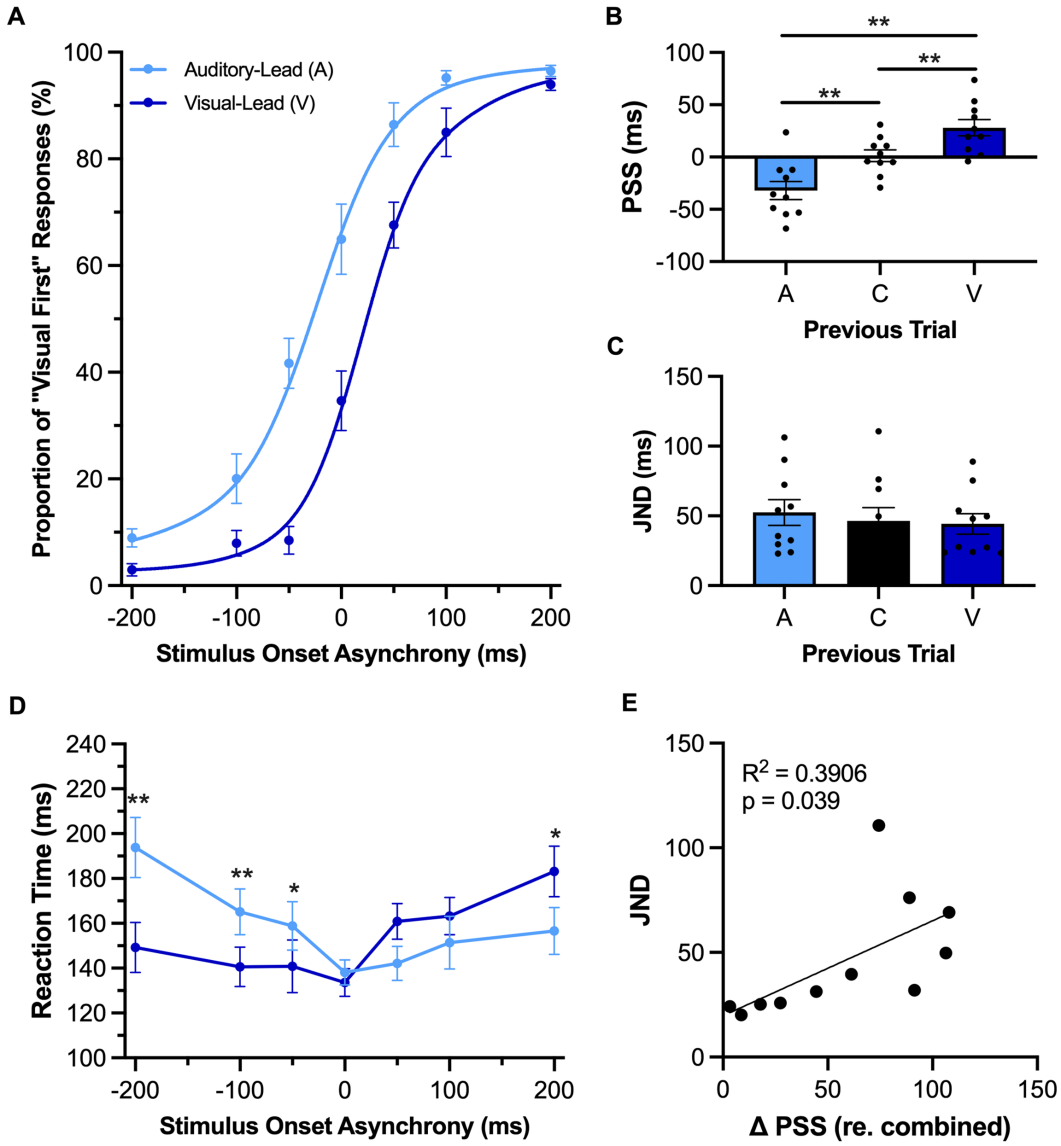
Rats showed evidence of rapid recalibration to asynchronous stimuli while performing the TOJ task. **(A)** Behavioral performance of Sprague Dawley rats (*n* = 11) on a temporal order judgment (TOJ) task plotted as proportion of “visual first” trials. Trials were separated into two categories, fitted then plotted: trials that followed an auditory-leading trial (light blue) or ones that followed a visual-leading trial (dark blue). **(B)** The point of subjective simultaneity (PSS) was extracted from the auditory-leading curve (A), visual-leading curve (V), and the combined curve which includes all trial types (C). As expected, the PSS was shifted in the direction of the previous trial. **(C)** The just noticeable difference (JND) of auditory-leading (A), combined trials (C), or visual-leading (V) curves did not change in response to the previous trial. **(D)** Reaction time of rats for each SOA for both auditory-leading (light blue) and visual-leading (dark blue) trials. **(E)** Relationship between the degree of change in PSS and the width of the JND, with the plotted line representing the liner regression fit. The change in PSS between visual-leading (V) and auditory-leading (A) trials were positively correlated with the width of the JND. Values are presented as mean ± SEM for PSS and TBW, *p < 0.05, **p < 0.0167 for **(B)** and **(C)**, *p < 0.05. ** < 0.0071 for **(D)**.

**Figure 4:**
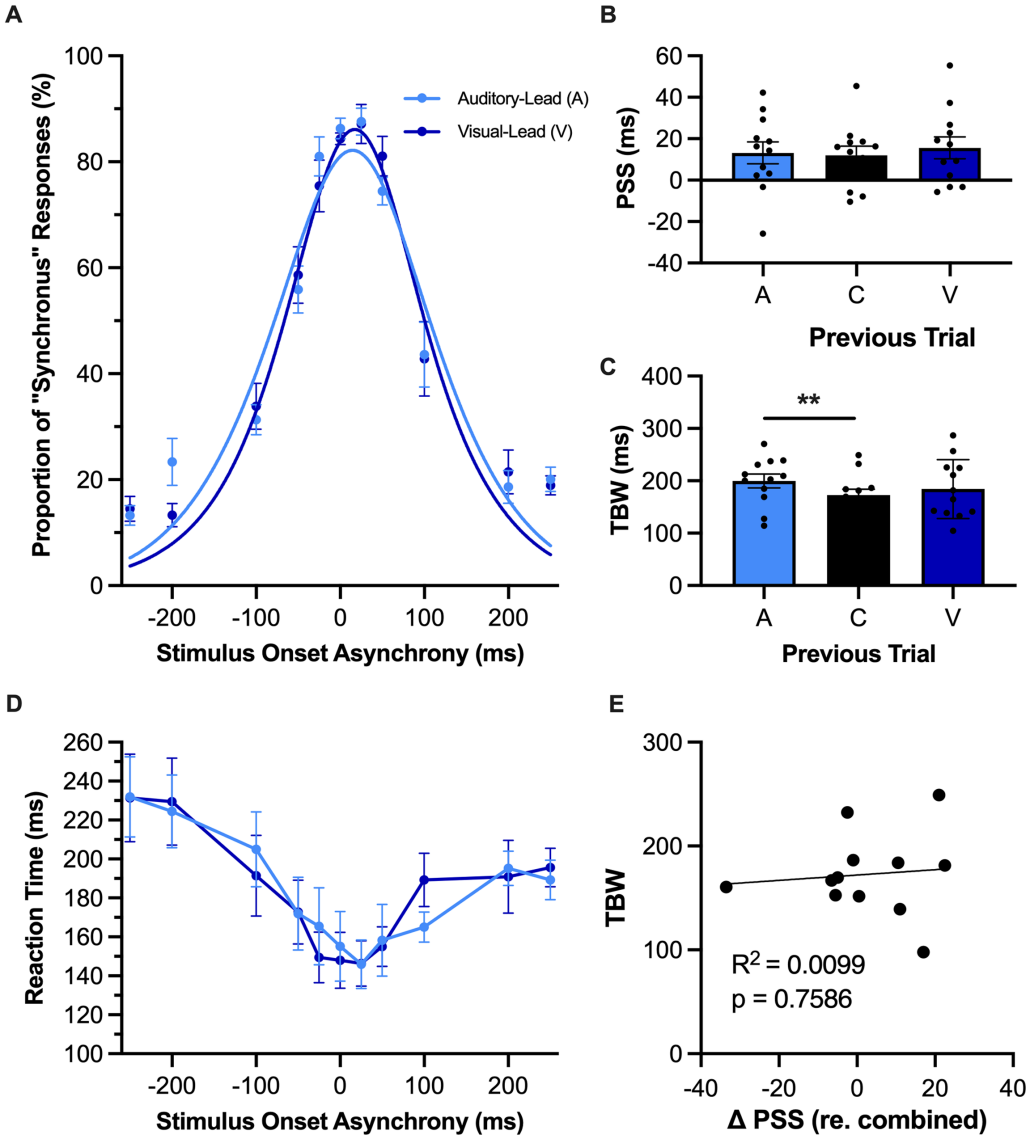
Rapid recalibration was not observed in rats performing the SJ task. **(A)** Behavioral performance of Sprague Dawley rats (*n* = 12) on a synchrony judgment (SJ) task plotted as proportion of “synchronous” trials. Trials were separated into two categories, fitted then plotted: trials that follow an auditory-leading trial (light blue) or ones that follow a visual-leading trial (dark blue). **(B)** The point of subjective simultaneity (PSS) was extracted from the auditory-leading curve (A), visual leading curve (V), and the combined curve which includes both trial types and synchronous trials (C). Unexpectedly, the rats performing the SJ task did not demonstrate rapid recalibration, as there was no shift in their PSS according to the preceding trial type. **(C)** The temporal binding window (TBW) of the three fitted curves, in which the TBW of auditory-leading (A) trials was significantly larger than the TBW of the combined curve (C), with no effect observed for visual-leading (V) TBW. **(D)** Reaction time of rats for each SOA for both auditory-leading (light blue) and visual-leading (dark blue) trials. Reaction time was not affected by the preceding trial. **(E)** For each rat, the degree of change in PSS was plotted against its TBW, with the line representing the liner regression fit. No correlation was observed for the change in PSS between visual-leading (V) and auditory-leading (A) trials and the TBW of the combined curve (C). Values are presented as mean ± SEM for PSS and TBW, *p < 0.05, **p < 0.017.

To examine whether rats, like humans, rapidly recalibrate their audiovisual temporal perception from one trial to the next during a test session, we computed their single-trial recalibration during performance of the TOJ and SJ tasks. To achieve this, a single-trial recalibration analysis was completed to separate trials into two categories; trials that followed an auditory-leading stimulus, or trials that followed a visual-leading stimulus. Trials for each test session were separated and fit to extract the PSS for both tasks, as well as the JND and TBW for the TOJ and SJ tasks, respectively. After fitting the functions, auditory-leading and visual-leading curves were plotted for both the TOJ task (Figure 3A) and SJ task (Figure 4A).

As can be seen in Figure 3A, the preceding trial had an influence on the rats’ perceptual judgments during the TOJ task. As expected, a one-way repeated-measures ANOVA found a significant main effect of preceding trial on the PSS [*F* (1.1, 10.7) = 22.85, *p* < 0.001, *ηp*^2^ = 0.70] (Figure 3B). More specifically, the PSS calculated for those trials which followed auditory-leading trials was significantly decreased when compared to the combined trials (A: −26.5 ± 8.5 ms vs. C: 4.0 ± 5.8 ms, *p* < 0.001), whereas the opposite pattern occurred following visual-leading trials, as the PSS significantly increased (V: 31.0 ± 7.7 ms vs. C: 4.0 ± 5.8 ms, *p* = 0.001). Despite these bidirectional shifts in the rats’ PSS, the preceding trial did not affect their temporal acuity during TOJ task performance, as there was no difference in JND between the conditions [*F* (2, 20) = 1.25, *p* = 0.31, *ηp*^2^ = 0.11, one-way repeated-measures ANOVA] (Figure 3C). In addition to examining the rapid recalibration of the rats’ audiovisual temporal perception, we also investigated whether they showed reaction time differences based on the leading stimulus modality of the preceding trial. A two-way repeated-measures ANOVA found a significant interaction of preceding trial by SOA [*F* (2.1, 21.1) = 8.92, *p* = 0.001, *ηp*^2^ = 0.47]; findings which suggest that the rats’ reaction time during the current trial was influenced by whether the audiovisual stimulus in the preceding trial was auditory-leading or visual-leading. Indeed, Bonferroni-adjusted post-hoc paired samples *t*-tests revealed that the reaction time on the trials which followed auditory-leading trials was significantly slower compared to the visual-leading trials at an SOA of −200 ms (*p* = 0.004) and − 100 ms (*p* = 0.003), whereas the rats’ reaction time was faster at an SOA of 200 ms (*p* = 0.04) (Figure 3D). In practical terms, these collective results suggest that if the rats performing the TOJ task were to be presented two successive trials of auditory-leading stimuli (e.g., A100V, then A200V), the first trial would cause the rats’ temporal perception to rapidly shift such that they would be more likely to judge the second trial as being visual-leading (see Figure 3A at −200 SOA), and this judgment would likely occur with a slower reaction time (see Figure 3D at −200 SOA).

In contrast to the significant shifts observed in the psychometric curves during the TOJ task (Figure 3A), there was unexpectedly no effect of the preceding trial on rats’ audiovisual temporal perception during the SJ task (Figure 4A) as the PSS calculated from the psychometric curves associated with the auditory-leading versus visual-leading trials did not significantly differ [*F* (2, 22) = 0.53, *p* = 0.59, *ηp*^2^ = 0.05, one-way repeated-measures ANOVA] (Figure 4B). Surprisingly, a one-way repeated-measures ANOVA did, however, reveal a main effect of the preceding trial type on the rats’ TBW [*F* (2, 22) = 0.53, *p* = 0.03, *ηp*^2^ = 0.28]. Furthermore, Bonferroni-adjusted paired samples *t*-tests found that the auditory-leading TBW was significantly wider compared to the combined TBW (199.8 ± 13.2 ms vs. 172.7 ± 11.5 ms, *p* = 0.009) (Figure 4C), whereas no difference was observed between the visual-leading TBW and the combined TBW (184.2 ± 16.7 ms vs. 172.7 ± 11.5 ms, *p* = 0.22). Finally, as can be seen in Figure 4D, there were no differences in the reaction times of rats performing the SJ task based on whether the preceding trial was auditory-leading or visual-leading (i.e., two-way repeated-measures ANOVA: no main effect of preceding trial type, [*F* (1, 11) = 0.05, *p* = 0.83, *ηp*^2^ < 0.01]; no interaction between preceding trial type & SOA, [*F* (4.2, 46.3) = 0.93, *p* = 0.46, *ηp*^2^ = 0.08]).

Due to the unexpected absence of rapid recalibration in rats performing the SJ task, we further investigated whether this may have been due to the large number of synchronous trials that were included in our experimental testing paradigm; ~ 1/3 of trials within an experimental test session were synchronous. To address this potential issue, we modified the SJ testing paradigm to have a reduced number of synchronous trials (~1/6 of trials) and then retested the same rats (*n* = 12). Similar to what was reported in Figure 4B, there were no changes in the PSS between auditory-leading and visual-leading psychometric curves derived from the modified SJ testing paradigm [i.e., one-way repeated-measures ANOVA: *F* (2, 12) = 0.75, *p* = 0.49, *ηp*^2^ = 0.11; auditory-leading PSS: 21.1 ± 5.3 ms vs. combined PSS: 20.7 ± 5.8 ms vs. visual-leading PSS: 25.5 ± 4.5 ms].

As a previous study on humans reported that the TBW may be predictive of the degree of change in the PSS (Van der Burg et al., 2013), we investigated whether rats experience a similar relationship between the temporal acuity and trial-by-trial adaptation of their audiovisual perception. Perhaps not surprising given the lack of rapid recalibration observed in the SJ task of the present study, there was no correlation between the shift in PSS and the TBW of the combined trials (*R*^2^ = 0.01, *p* = 0.76) (Figure 4E). Conversely, in the TOJ task (Figure 3E), we did observe a positive correlation between the shift in the PSS (visual-leading minus auditory-leading) and the JND derived from the combined trials, where a larger JND was correlated with a greater shift in PSS between visual-leading and auditory-leading trials (*R*^2^ = 0.39, *p* = 0.04). Overall, these correlative findings align with the other results of Experimental Series 2 to suggest that rats, like humans, can experience a rapid recalibration of their audiovisual temporal perception that occurs from one trial to the next during a test session; however, this phenomenon was only evident when the rats performed the TOJ task, not the SJ task.

### 3.3. Decreasing the signal-to-noise ratio of auditory stimulation predictably altered the rats’ audiovisual temporal perception during performance of the TOJ task

**Figure 5:**
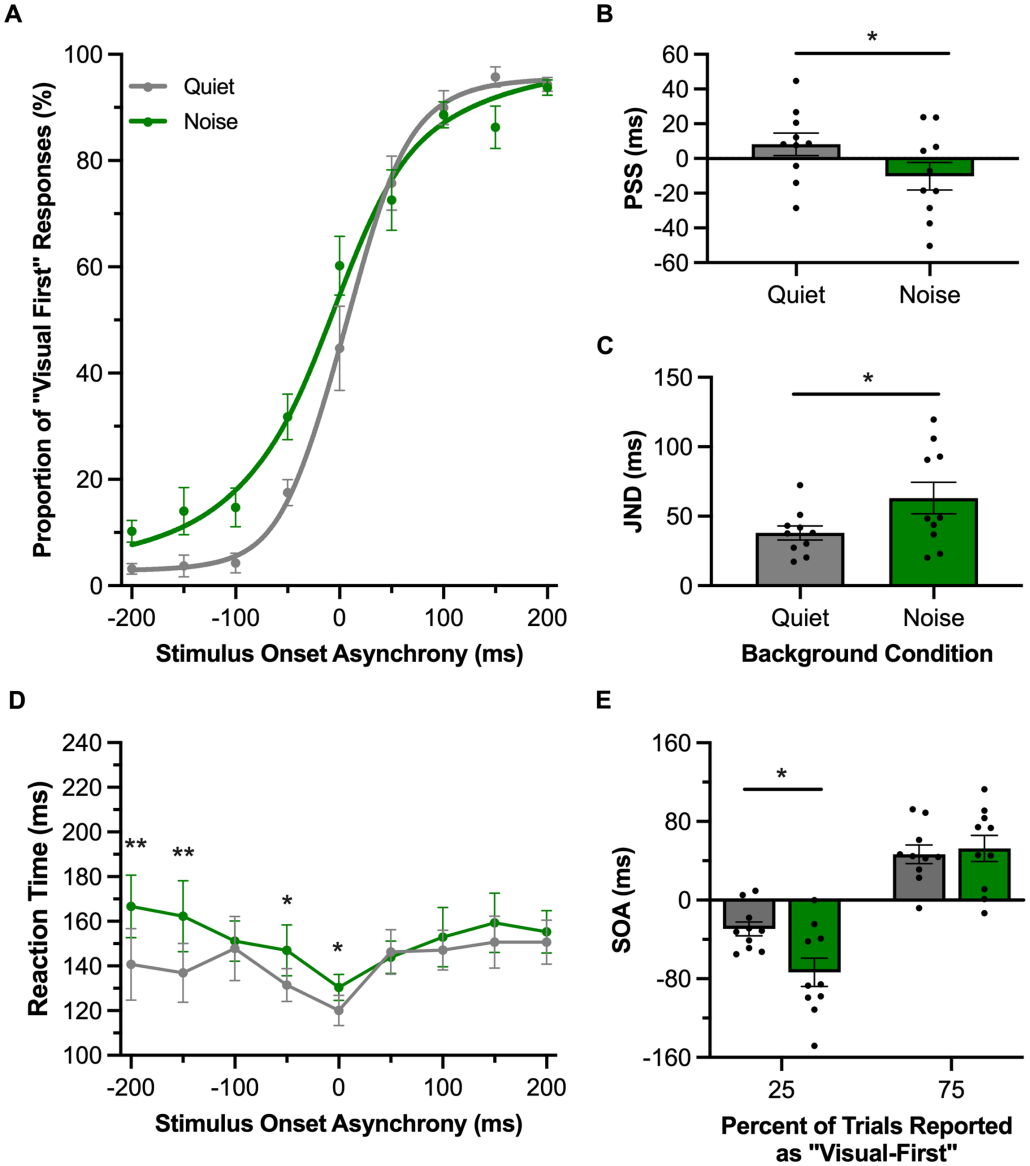
Background noise altered audiovisual temporal perception in rats performing the TOJ task. **(A)** Behavioral performance of Sprague Dawley rats (*n* = 10) on a temporal order judgment (TOJ) task plotted as proportion of “visual first” trials. Rats were either tested with a 60 dB SPL background noise played from a second speaker (Noise; green) or with no added background noise (Quiet; gray). **(B)** The point of subjective simultaneity (PSS) shifted to the left side of the psychometric curve in response to background noise. **(C)** The just noticeable difference (JND) of the fitted curves widened in response to background noise. **(D)** Reaction time of rats for each SOA for the background noise (green) or quiet control (gray) testing conditions. **(E)** The bounds of the JND values at the 25 and 75% proportions of “visual-first” responses. The left bound, but not the right, was significantly widened in response to background noise. Values are presented as mean ± SEM for PSS and TBW, *p < 0.05, **p < 0.0046.

In Experimental Series 3, rats underwent their behavioral testing under normal (quiet) conditions and in the presence of a 60 dB SPL background noise to ultimately determine if this decrease in the signal-to-noise ratio of the auditory stimulation would affect their temporal acuity, especially with respect to judging the temporal order of the stimuli in auditory-leading trials. For the TOJ task, the rats’ performance during the two experimental test sessions were fit with sigmoidal functions and plotted (Figure 5A). In the presence of background noise, there was a significant decrease in the PSS, such that the psychometric curve shifted leftward (i.e., shifted towards the auditory-leading side, *p* = 0.02, paired samples *t*-test) (Figure 5B). Moreover, background noise not only influenced the rats’ perception of the order of the auditory and visual stimuli, but it also impaired their temporal acuity, as there was a significant increase in JND when compared to the quiet testing condition (*p* = 0.01, paired samples *t*-test) (Figure 5C). We further examined this noise-induced decrease in temporal acuity by comparing the rats’ performance at the bounds of the JND (i.e., 25 and 75%), to ultimately determine whether there was a consistent effect on both sides of the psychometric curve. A two-way repeated-measures ANOVA found a significant interaction of testing condition (i.e., background noise vs. quiet) by JND values (i.e., 25% vs. 75%) [*F* (1, 9) = 9.72, *p* = 0.01, *ηp*^2^ = 0.52]. Subsequent analyses using Bonferroni-adjusted post-hoc paired samples *t*-tests revealed that only the left side of the JND (i.e., 25%) showed a significant change in the presence of background noise (*p* = 0.02; Figure 5E); findings consistent with our prediction that a decrease in the signal-to-noise ratio of the auditory stimulation would preferentially impair the rats’ ability to accurately judge the temporal order of the audiovisual stimuli on auditory-leading trials. In addition to assessing the consequence of the background noise on the temporal acuity of the rats’ audiovisual perception, we also examined the effect on the rats’ reaction time across a range of SOAs. A two-way repeated-measures ANOVA found a significant interaction of testing condition by SOA [*F* (3.4, 30.9) = 3.66, *p* = 0.02, *ηp*^2^ = 0.29] (Figure 5D), and Bonferroni-adjusted paired samples *t*-tests showed that, in the presence of background noise, rats had a significantly slower reaction time with auditory-leading stimuli (SOA of −200 ms, *p* = 0.004; −100 ms, *p* = 0.001).

**Figure 6:**
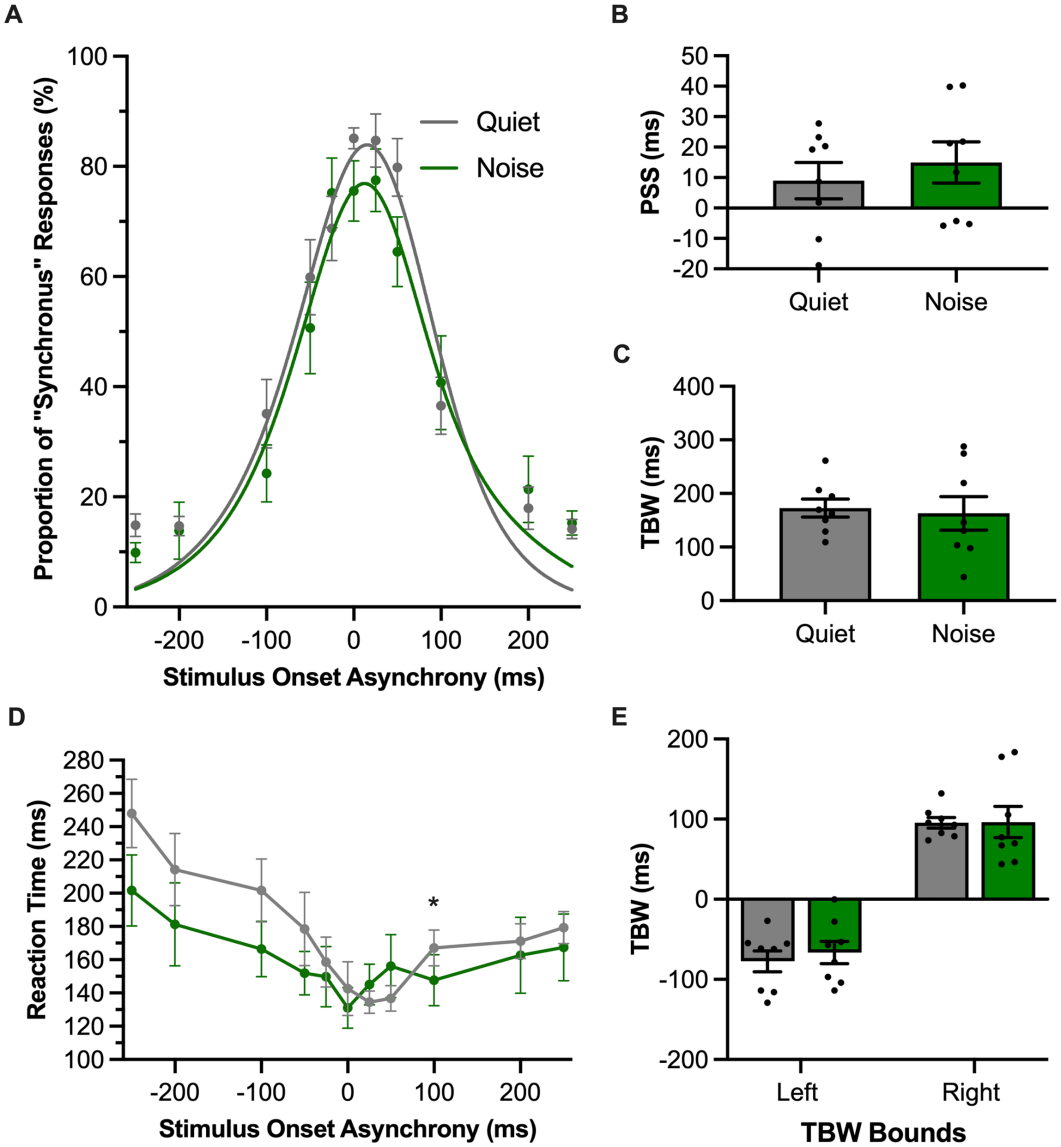
Background noise did not significantly alter audiovisual temporal perception in rats performing the SJ task. **(A)** Behavioral performance of Sprague Dawley rats (*n* = 8) on a synchrony judgment (SJ) task plotted as proportion of “synchronous” trials. Rats were either tested with a 60 dB SPL background noise played from a second speaker (Noise; green) or with no added background noise (Quiet; gray). **(B,C)** The point of subjective simultaneity (PSS; panel **B**) and the temporal binding window (TBW; panel **C**) of the fitted curves did not shift in response to background noise. **(D)** Reaction time of rats for each SOA for the background noise (green) or quiet control (gray) testing conditions. **(E)** Assessment of the bounds of the TBW values at the 50% perceived synchronous trials from the center of the fitted curve revealed no change in response in the background noise versus quiet condition. Values are presented as mean ± SEM for PSS and TBW, *p < 0.05.

In the SJ task, rats reported whether they perceived the audiovisual stimulation to have been synchronous or asynchronous in the presence of a 60 dB SPL background noise or the standard (quiet) testing condition. For each of these experimental test sessions, performance across all trials was fit with a gaussian function and plotted as the proportion of trials perceived as synchronous (Figure 6A). In contrast to the TOJ task, the presence of the background noise did not affect the PSS in the SJ task (*p* = 0.39, paired samples *t*-test; Figure 6B) or the TBW (*p* = 0.73, paired samples *t*-test; Figure 6C) compared to the quiet condition. We further examined whether there were differences in the TBW on the left vs. right sides of the psychometric curves between the quiet and background noise conditions. A two-way repeated-measures ANOVA revealed no interaction between the sides of the psychometric curve and the testing condition [*F* (1, 7) = 0.13, *p* = 0.73, *ηp*^2^ = 0.02] (Figure 6E). Lastly, we examined whether background noise influenced the rats’ time to react to the audiovisual stimuli during the SJ task. A two-way repeated-measures ANOVA found a significant interaction of testing conditions by SOA [*F* (3.8, 26.7) = 3.66, *p* = 0.02, *ηp*^2^ = 0.34] (Figure 6D). Overall, the collective results of Experimental Series 3 found that the presence of background noise differentially affected the rats’ performance in the two audiovisual perceptual judgment tasks, such that only the TOJ task showed evidence that the rats’ audiovisual temporal perception was altered by a decrease in the signal-to-noise ratio of the auditory stimulation.

### 3.4. Pharmacological disruption of glutamatergic neurotransmission worsened the rats’ ability to judge the timing of auditory and visual stimuli

**Figure 7:**
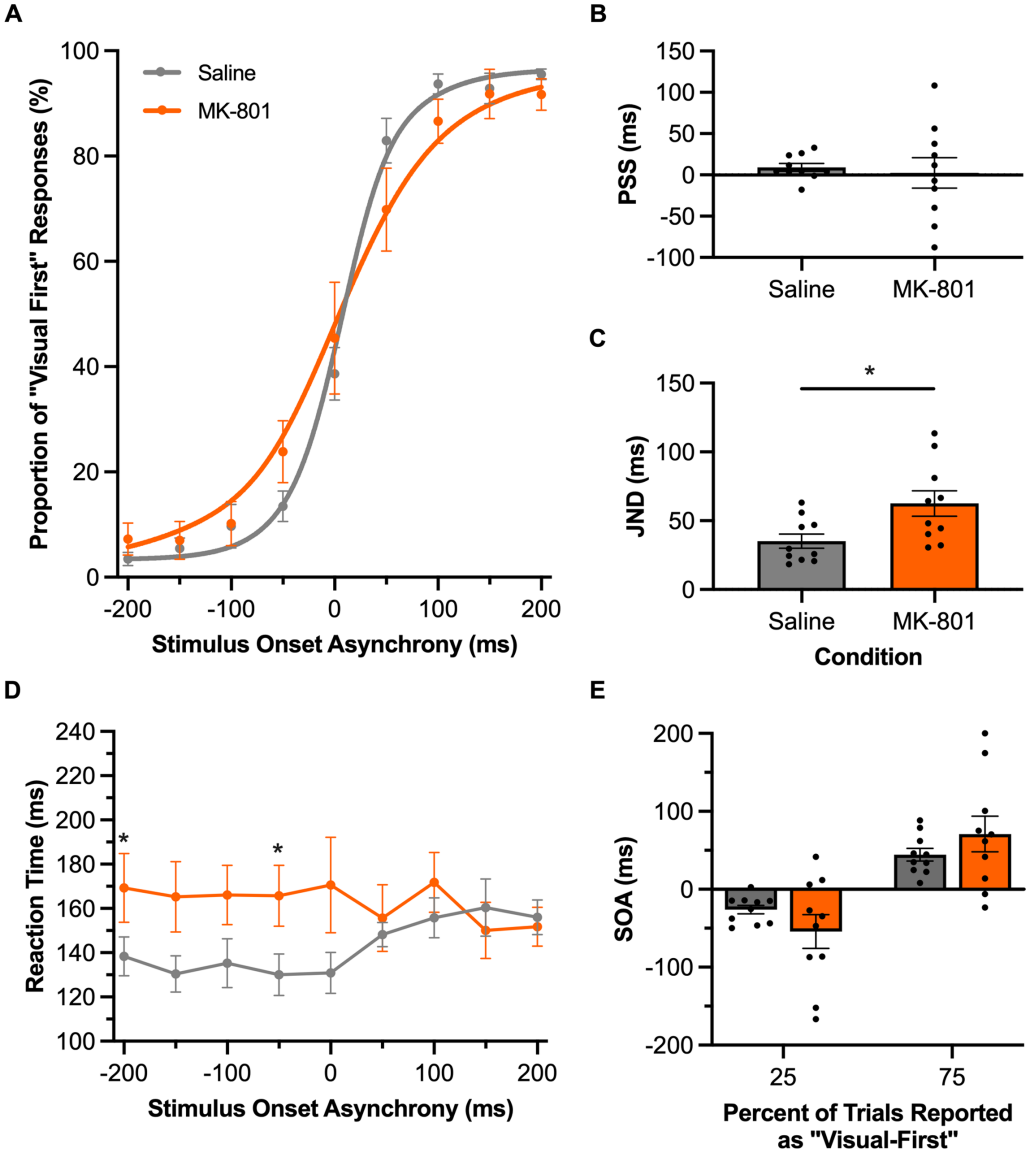
Disruption of glutamatergic neurotransmission impaired audiovisual temporal acuity in rats performing the TOJ task. **(A)** Behavioral performance of Sprague Dawley rats (*n* = 10) on a temporal order judgment (TOJ) task plotted as proportion of “visual first” trials. Rats were subcutaneously injected with MK-801 (0.1 mg/kg; orange) or a saline control (gray). **(B)** The point of subjective simultaneity (PSS) did not shift for the MK-801 treatment in comparison to the control treatment. **(C)** Compared to the control treatment, MK-801 caused an increase in the just noticeable difference (JND) of the fitted curves; evidence of a decrease in temporal acuity. **(D)** Reaction time of rats for each SOA for the MK-801 (orange) and control treatment (gray). **(E)** The bounds of the JND values at the 25 and 75% proportions of “visual-first” responses did not change between the two treatments. Values are presented as ± SEM for PSS and TBW, *p < 0.05.

In Experimental Series 4, we disrupted the rats’ glutamatergic neurotransmission via a subcutaneous injection of MK-801 and then assessed the consequences on their performance of the TOJ or SJ task. For the TOJ task, compared to the saline condition, systemic injection of MK-801 did not affect the group mean PSS value (*p* = 0.73, paired samples *t*-test); however, the performance results of each rat appeared to be more variable following MK-801 versus saline (Figure 7B). Furthermore, MK-801 caused the rats to have a larger JND than compared to the saline condition (*p* = 0.03, paired samples *t*-test; Figure 7C); findings which suggest that disruption in glutamatergic neurotransmission worsened the rats’ audiovisual temporal acuity (i.e., they had more difficulty judging the temporal order of the auditory and visual stimuli). We further examined the increased JND following MK-801 injection by comparing performance at the bounds of the JND to determine if the decreased temporal acuity was specific to the rats’ ability to judge the temporal order of auditory-leading stimuli (25% bound) or visual-leading stimuli (75% bound). Although a two-way repeated-measures ANOVA found a significant interaction of drug by JND bound [*F* (1,9) = 6.2, *p* = 0.03, *ηp*^2^ = 0.41], Bonferroni-adjusted post-hoc paired samples *t*-tests failed to reveal a specific effect of disrupting glutamatergic neurotransmission on either of the bounds of the JND (25%: *p* = 0.21; 75%: *p* = 0.35) (Figure 7E). Finally, compared to the saline injection, MK-801 affected the rats’ reaction times at specific SOAs, as a two-way repeated-measures ANOVA found a significant interaction between the drug injected and SOA [*F* (8,72) = 2.43, *p* = 0.02, *ηp*^2^ = 0.21] and Bonferroni-adjusted paired samples *t*-tests revealed a trend for the rats to have slower reaction times during all of the SOAs when the auditory stimulus preceded the visual stimulus (i.e., A200V to A50V; *p*-values ranged from 0.03 to 0.07) (Figure 7D).

**Figure 8:**
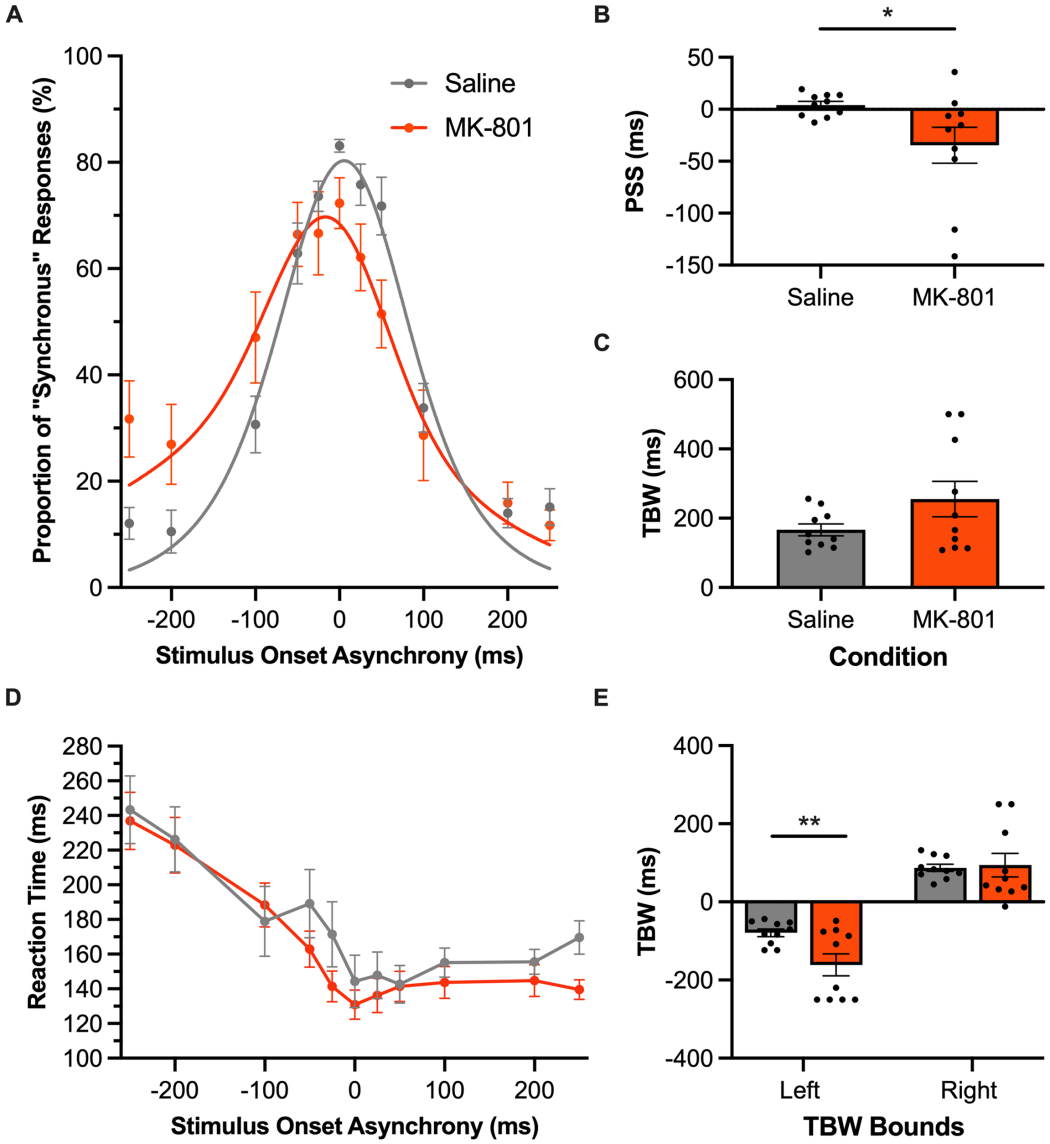
Audiovisual temporal acuity, specifically during auditory-leading trials of the SJ task, was impaired following disruption of glutamatergic neurotransmission. **(A)** Behavioral performance of Sprague Dawley rats (*n* = 8) on an SJ task plotted as proportion of “synchronous” trials. Rats were subcutaneously injected with MK-801 (0.1 mg/kg; orange) or a saline control (gray). **(B)** The point of subjective simultaneity (PSS) shifted in the MK-801 treatment compared to the control treatment. **(C)** The temporal binding window (TBW) calculated from the fitted curves did not significantly differ between the two treatment conditions (*p* = 0.08). **(D)** Reaction time of rats for each SOA for the MK-801 (orange) and control treatment (gray). **(E)** MK-801 significantly changed the left bound of the TBW compared to the control treatment; suggesting that MK-801 exerted a greater effect on the rats’ ability to judge asynchronous stimuli when the auditory stimulus preceded the visual. Values are presented as mean ± SEM for PSS and TBW, *p < 0.05, **p < 0.017.

Inspection of the SJ task psychometric curves showed a leftward shift following MK-801 injection (Figure 8A), which corresponded to a significant decrease in the rats’ PSS compared to the saline condition (*p* = 0.045, paired samples *t*-test; Figure 8B). In addition to the rats more frequently judging auditory-leading stimuli to be synchronous (i.e., negative PSS), MK-801 injection also tended to worsen the rats’ temporal acuity, evidenced by a trend for their TBW to widen (MK-801: 255.3 ± 51.1 ms vs. saline: 166.5 ± 17.3 ms, *p* = 0.08, paired samples *t*-test; Figure 8C). Moreover, a two-way repeated-measures ANOVA found a significant main effect of the drug on the bounds of the TBW [*F* (1, 9) = 7.79, *p* = 0.021, *ηp*^2^ = 0.46], and Bonferroni-adjusted paired samples *t*-tests revealed a significant shift in the left TBW bound (*p* = 0.003) but not the right (*p* = 0.41). Taken together, these collective findings from the SJ task suggest that systemic disruption in glutamatergic neurotransmission preferentially affected the rats’ temporal acuity as they needed to accurately judge trials when the auditory stimulus preceded the visual stimulus. Finally, these perceptual changes caused by MK-801 injection were not accompanied by altered reaction times compared to the saline condition, as a two-way repeated-measures ANOVA did not find a significant main effect of drug [*F* (1, 9) = 1.58, *p* = 0.24, *ηp*^2^ = 0.15] nor a significant interaction between the drug injected and SOA [*F* (3.4, 30.5) = 1.74, *p* = 0.18, *ηp*^2^ = 0.16].

## 4. Discussion

Toward the eventual goal of using animal research to uncover the cellular and molecular mechanisms of audiovisual temporal perception, there is a need to first establish the translational potential of any prospective animal model by ensuring that they show evidence of the hallmarks of audiovisual temporal perception reported in human studies. Guided by the testing paradigms performed on human participants, previous animal studies have confirmed that rats can be trained to report the temporal order of auditory and visual stimuli, with performance metrics fairly consistent with humans (Schormans et al., 2017; Mafi et al., 2023; Paulcan et al., 2023). In the present study, we extended this past work by first developing an audiovisual synchrony judgment (SJ) task for rats that could assess their temporal binding window (i.e., the epoch of time over which they had a high likelihood of perceiving the auditory and visual stimuli as being synchronous). Then, over a series of experiments, we used this new SJ task and a previously established temporal order judgment (TOJ) task to show that rats, like humans, can experience malleability of their audiovisual temporal perception based on their past and present sensory experiences (Experimental Series 1 & 2, respectively). Furthermore, by manipulating either the testing conditions with a background noise (Experimental Series 3) or altering the rats’ neurochemistry via pharmacological antagonism of glutamatergic neurotransmission (Experimental Series 4), we assessed the extent that the TOJ and SJ tasks could identify when the rats had perceptual difficulty judging the timing of the auditory and visual stimuli. Overall, our collective findings provide strong support for using rats for future studies into the mechanistic basis of audiovisual temporal perception and its disruption in clinical conditions.

### 4.1. The influence of past & present experience on audiovisual temporal perception

In the present study, we reasoned that if rats are to effectively serve as an animal model for studying audiovisual temporal perception they should demonstrate experience-dependent perceptual shifts that would be predicted from human studies. When quantifying this malleability in a participants’ audiovisual temporal perception, the metric of interest—the point of subjective simultaneity (PSS)—can be calculated from the psychometric curves of both the TOJ and SJ tasks; however, due to the inherent differences between the tasks, the associated PSS represents nuanced aspects of the participant’s perception (van Eijk et al., 2008). For the TOJ task, the PSS identifies the relative timing of the auditory and visual stimuli when the participant was most unsure of the temporal order, whereas in the SJ task the PSS quantifies the timing of the auditory and visual stimuli that had the highest chance of being perceived as synchronous, even if the stimuli were not. Because a given participant’s PSS can differ when they perform a TOJ versus SJ task (van Eijk et al., 2008) it was important for us to first develop a complete version of the SJ task for rats before ultimately determining whether they, like humans, experience a shift in their PSS depending on prior sensory experiences. As shown in Figure 2B, we found that the rats’ PSS extracted from the psychometric curves of the newly-developed SJ task were slightly rightward shifted (i.e., the rats’ perceived synchrony when the visual stimulus preceded the auditory by 12 ms); an observation that is consistent with both human behavioral studies (Zampini et al., 2005a; van Eijk et al., 2008; Vroomen and Keetels, 2010) as well as animal electrophysiological data (Meredith et al., 1987; Wallace et al., 1996; Schormans and Allman, 2019; Schormans and Allman, 2023).

It is well established that repeated exposure to asynchronous audiovisual stimuli can predictably shift humans’ audiovisual temporal perception of simultaneity toward the direction of the asynchronous pairing (Fujisaki et al., 2004; Vroomen et al., 2004; Navarra et al., 2005; Vatakis et al., 2008b; Roseboom and Arnold, 2011; Heron et al., 2012; Roseboom et al., 2013; O’Donohue et al., 2022). This perceptual malleability is bidirectional, as passive exposure to an auditory-leading stimulus pair causes a leftward shift in the participant’s psychometric curve, resulting in a more negative PSS value, whereas the opposite shift occurs when the passive exposure is a visual-leading stimulus pair. As seen in Figures 1, 2, when the rats were presented asynchronous audiovisual stimulation prior to performing the TOJ and SJ tasks, they, too, showed predictable shifts in their psychometric curves and resultant PSS values; findings which represent the first time that this form of audiovisual perceptual malleability has been reported in non-human subjects.

Consistent with recent studies (Scott et al., 2020; Schormans and Allman, 2023), we confirmed that rats performing the TOJ task also show evidence of a rapid recalibration of their temporal perception that depends on the timing lag between the auditory and visual stimuli in the preceding trial (Figure 3). As expected, the rats experienced the characteristic profile of rapid recalibration; e.g., when the current trial was a synchronous audiovisual stimulus, and the preceding trial had been a visual-leading pairing, the rats were more likely to judge the current trial as being auditory-leading, not synchronous. Based on these TOJ task results, we were surprised that the rats performing the newly-developed SJ task did not demonstrate rapid recalibration of their audiovisual temporal perception (Figure 4). In search of a possible methodological basis for this unexpected result, we carried out a supplemental experiment to determine whether the high number of synchronous trials included in the SJ task prevented the emergence of rapid recalibration. Ultimately, despite having the rats perform a modified version of the SJ task with far fewer synchronous trials, we still did not observe recalibration of their audiovisual temporal perception from one trial to the next.

In speculating as to the reason why rats performing the SJ task failed to show an influence of the preceding trial on their current temporal perception, it is important to not only consider possible methodological issues, but to also contemplate whether the rats’ decisional strategy during task performance may have precluded the emergence of rapid adaptation. Based on SJ task-related EEG data and modeling analyses in humans, Simon et al. (2017, 2018) suggested that rapid recalibration of audiovisual temporal perception arises from decision-based factors, rather than early-sensory mechanisms. Because the participants’ reaction times were inherent in the modeling analyses that supported Simon and colleagues’ assertion, one might expect that if the rats in the present study were indeed relying on a performance strategy that differed from humans, they would also show a differential profile of reaction times across the SOAs of the SJ task. Interestingly, this was not the case, as both species showed a similar reaction time profile, in which the rats (Figure 4D) and humans (see Figure 1D in Simon et al., 2018) had longer reaction times for the judgments made at the largest temporal asynchronies (e.g., at the extremes of the SOAs: A250V and V250A; Figure 4D) compared to when the auditory and visual stimuli were presented synchronously. As noted by Simon et al. (2017), this profile of reaction times over the varying SOAs could perhaps be explained by previous fMRI findings which showed increased processing activity at greater levels of asynchrony (Stevenson et al., 2010). As it stands, additional research is necessary to discern why it was that the rats experienced rapid recalibration of their audiovisual temporal perception in the TOJ task yet failed to show evidence of this phenomenon in the SJ task, despite having a reaction time profile consistent with humans.

Overall, the collective results of Experimental Series 1 and 2 show that rats performing the TOJ task experienced malleability of their audiovisual temporal perception consistent with humans following both prolonged and recent exposure to asynchronous stimuli. In addition to experiencing these predictable shifts in their PSS (Figures 1C, 3B), the rats, like humans (Harrar and Harris, 2008), also showed that the passive exposure protocol did not impact their temporal acuity during the TOJ task (i.e., their JND was unaffected; Figure 1D). Finally, we observed a positive relationship between the rats’ temporal acuity and the magnitude of their rapid recalibration (measured as the change in PSS; Figure 3E); findings which are consistent with past reports in which the participants with wider windows of temporal integration experience larger perceptual shifts from one trial to the next (Van der Burg et al., 2013; Simon et al., 2017, 2018; Han et al., 2022). Given that different neural mechanisms are thought to underlie the perceptual malleability associated with prolonged versus recent exposure to asynchronous audiovisual stimuli (Van der Burg et al., 2015a; Simon et al., 2017; Van der Burg et al., 2018), we can envision future studies using a combination of electrophysiological recordings and opto/chemogenetic manipulations in rat models to ultimately investigate the neural mechanisms supporting the malleability of audiovisual temporal perception.

### 4.2. Sensory-specific effects on audiovisual temporal perception

In Experimental Series 3, we observed that a competing background noise caused a leftward shift of the rats’ PSS and decreased their temporal acuity (i.e., JND increased) during the TOJ task (Figure 5), with no effect on the rats’ PSS and TBW derived from the psychometric curves of the SJ task (Figure 6). Whereas past studies on humans also showed that TOJ task performance is sensitive to the intensity of the auditory and visual stimuli (Smith, 1933; Neumann et al., 1992; Boenke et al., 2009), to our knowledge no prior research has investigated the effect of altering sound intensity on participants’ PSS and TBW derived from a full version of the SJ task which includes physically-synchronous stimuli as well as auditory-leading and visual-leading stimuli. That said, Smith (1933) reported that manipulating the sound intensity during an SJ task had limited effect on the participants’ threshold to detect simultaneity, whereas it significantly altered their TOJ task performance (as measured by the reaction times needed to make correct temporal judgments). Overall, given that previous studies have highlighted important behavioral (Mossbridge et al., 2006; van Eijk et al., 2008) and neurophysiological (Binder, 2015; Basharat et al., 2018; Love et al., 2018) differences between performance of TOJ versus SJ tasks, perhaps it is not too surprising that we observed a differential effect between the two tasks when the rats performed them in the presence of a background noise.

### 4.3. Assessing deficits in the rats’ temporal acuity using the TOJ and SJ tasks

In the present study, we were motivated to determine if the TOJ and SJ tasks could identify when rats had perceptual difficulty judging the timing of the auditory and visual stimuli. To that end, we assessed the extent that altering the rats’ neurochemistry via pharmacological antagonism of glutamatergic neurotransmission affected their audiovisual temporal acuity (Experimental Series 4). As predicted, systemic injection of MK-801 worsened the rats’ JND and TBW during the TOJ and SJ tasks, respectively. Interestingly, MK-801 seemed to preferentially impact task performance related to auditory stimulation. For example, as seen in Figure 8, following the MK-801 injection, the rats’ TBW was widened in the SJ task due to a leftward shift in the psychometric curve, indicative of the rats’ experiencing a greater difficulty judging asynchronous stimuli when the auditory stimulus preceded the visual (e.g., −200 ms SOA in Figure 8A). Moreover, for the TOJ task, MK-801 injection increased the rats’ reaction times preferentially during the auditory-leading trials (Figure 7D). Interestingly, although not much is known about the effects of MK-801 on the visual system, numerous studies have shown that it not only causes significant disruption to auditory processing, as measured by sound-evoked cortical oscillations and event-related potentials (Sivarao et al., 2013; Parras et al., 2020; Janz et al., 2022), but MK-801 also causes deleterious effects on higher-level cognitive function (e.g., decision-making and attention; Paine and Carlezon, 2009; Onofrychuk et al., 2021; Patrono et al., 2023). Thus, it is possible that the MK-801 injection altered task performance in the present study by impairing the rats’ ability to process the auditory stimuli and/or by altering their decision strategies. Although additional experiments are required to directly test these theories, our findings highlight that the TOJ and SJ tasks were indeed able to reveal specific performance deficits when the rats had difficulty detecting the subtle timing differences between the auditory and visual stimulation. Overall, in considering these MK-801 results in combination with the collective findings from the other experimental series, we conclude that rats represent an excellent animal model to further investigate the neural basis of audiovisual temporal perception as well as the cellular and molecular mechanisms underlying alterations in its acuity and malleability that are commonly reported in various clinical conditions.

## Data availability statement

The raw data supporting the conclusions of this article will be made available by the authors, without undue reservation.

## Ethics statement

The animal study was approved by University of Western Ontario Animal Care and Use Committee. The study was conducted in accordance with the local legislation and institutional requirements.

## Author contributions

MA: Writing – original draft, Writing – review & editing, Conceptualization, Methodology. AS: Writing – original draft, Writing – review & editing, Conceptualization, Methodology, Software, Supervision. BA: Writing – original draft, Writing – review & editing, Conceptualization, Funding acquisition, Supervision.
